# The protective role of cannabidiol in stress-induced liver injury: modulating oxidative stress and mitochondrial damage

**DOI:** 10.3389/fphar.2025.1567210

**Published:** 2025-03-14

**Authors:** Chengyu Huang, Huichao Liang, Xiaohua Liang, Yueyi Liu, Jiaoling Wang, Haoran Jiang, Xinhui Kou, Jun Chen, Lili Huang

**Affiliations:** ^1^ Department of Pharmacy, Shenzhen Traditional Chinese Medicine Hospital, Shenzhen, China; ^2^ School of Pharmacy, Guangdong Pharmaceutical University, Guangzhou, China; ^3^ The Fourth Clinical Medical College, Guangzhou University of Chinese Medicine, Guangzhou, China

**Keywords:** stress-induced liver injury, differential gene expression analysis, mitochondria, cannabidiol, oxidative stress

## Abstract

**Background:**

Stress-induced liver injury, resulting from acute or chronic stress, is associated with oxidative stress and inflammation. The endocannabinoid system, particularly cannabinoid receptor 2 (CB_2_R), plays a crucial role in liver damage. However, there are currently no clinical drugs targeting CB_2_R for liver diseases. Cannabidiol (CBD), a CB2R agonist, possesses anti-inflammatory and antioxidant properties. This study aims to investigate the pharmacological effects of CBD in a mouse model of stress-induced liver injury.

**Methods:**

We employed a mouse model of stress-induced liver injury to evaluate the protective effects of CBD. Assessments included histopathological analysis, cytokine detection via ELISA, protein expression analysis using immunohistochemistry and Western blot, and gene transcription differential analysis. Transmission electron microscopy was utilized to observe mitochondrial morphology. Additionally, we examined the expression levels of CB_2_R, SLC7A11, α-SMA, and ACSL4 proteins to elucidate the mechanisms underlying CBD’s effects.

**Results:**

CBD exhibited significant protective effects against stress-induced liver injury in mice. Decreases in liver function indicators (including Aspartate Aminotransferase (AST) and Alanine Aminotransferase (ALT)) and inflammatory cytokines (such as IL-1β and Tumor Necrosis Factor-alpha (TNF-α)) were observed. CBD enhanced CB_2_R expression and reduced α-SMA levels, mitigating liver fibrosis. It also decreased ACSL4 levels, increased SOD and GSH-Px activities, and upregulated SLC7A11 protein expression. Furthermore, CBD improved mitochondrial morphology, indicating a reduction in oxidative cell death.

**Conclusion:**

CBD activates the CB_2_R/α-SMA pathway to modulate liver inflammation and fibrosis. Through the SLC7A11/ACSL4 signaling pathway, CBD alleviates oxidative stress in stress-induced liver injury, enhances mitochondrial morphology, and reduces liver damage. These findings provide a theoretical basis for the potential application of CBD in the prevention and treatment of stress-induced liver injury.

## Graphical Abstract

**Figure:**
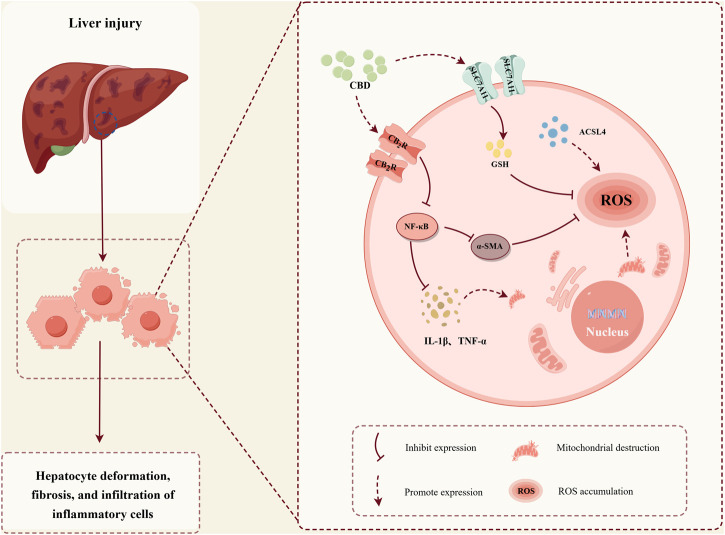
The protective role of CBD in stress-induced liver injury. (Image created with Figdraw, Export code: YTUOT76e0e).

## 1 Introduction

Stress reaction refers to the physiological and psychological responses of the body when exposed to stressors (Kim et al., 2019). The neuroendocrine response is closely linked to stress, primarily involving the sympathetic-adreno-medullary system and the hypothalamic-pituitary-adrenal (HPA) axis. In addition, hormones such as angiotensin are also involved in this response (Selye, 1998). Short-term stress helps the body adapt to environmental changes and maintain homeostasis. However, chronic stress can impair the body’s self-regulation and repair mechanisms, leading to multi-organ dysfunction and even death (Li et al., 2022). The liver is a crucial metabolic organ in the body responsible for detoxifying harmful substances (Kim et al., 2020), yet it is also highly susceptible to stress stimuli (Wang et al., 2016). Studies have shown that stress triggers a series of complex biological and behavioral responses, leading to the activation of the HPA axis and stimulation of the sympathetic nervous system, resulting in increased levels of adrenaline, cortisol, and pro-inflammatory cytokines (Schneiderman et al., 2005). All of these factors can induce liver injury. Acute stress-induced liver damage can lead to infections or shock, posing a serious threat to health and life (Chen et al., 2017).

However, in addressing the critical challenge of stress-induced liver injury, traditional pharmacological treatments, while somewhat effective, often fail to fully meet clinical needs. Agents such as polyene phosphatidylcholine, glutathione, and tiopronin are commonly used but are associated with adverse effects on the gastrointestinal and cardiovascular systems (Zhang, 2023), and in some cases, may even exacerbate liver injury (Dini et al., 2024). Therefore, it is of significant importance to identify more effective and safer hepatoprotective drugs.

Cannabidiol (CBD) is a natural compound derived from the cannabis plant and is widely present in cannabis essential oil. CBD exhibits various pharmacological activities, including anti-inflammatory, anxiolytic, and neuroprotective effects, and it is considered to have a relatively high safety profile (Basavarajappa and Subbanna, 2024). CBD has been assessed by the World Health Organization (WHO) as safe (Fitzcharles et al., 2023) and has been approved in several countries for the treatment of specific diseases (McGregor et al., 2020; Fiani et al., 2020). Research has shown that CBD can interact with cannabinoid receptors, particularly activating the cannabinoid receptor 2 (CB_2_R) (Thomas et al., 2007), and the activation of CB_2_R has been found to exert protective effects against liver injury (Teixeira-Clerc et al., 2006). Although preclinical studies suggest that selective CB_2_R agonists may be used for the treatment of liver diseases, no CB_2_R agonists are currently available for human trials. Unlike the potent synthetic CB_2_R agonists used in animal models, CBD is a naturally occurring CB_2_R agonist with multiple pharmacological effects and a relatively low risk of side effects, making it a promising candidate for the treatment of liver injury caused by various factors.

This study investigated the protective effects of CBD against stress-induced liver injury and revealed its pharmacological effects and underlying mechanisms. These findings provided further insights for the development of new drugs aimed at preventing and treating stress-induced liver injury.

## 2 Materials and methods

### 2.1 Reagents

CBD was purchased from Xi’an Xiaocao Plant Technology Co., Ltd., (Xi’an, China); Corticosterone (CORT, Cat. No. CSB-E07969m) ELISA Kit was purchased from Cusabio (Wuhan, China); Tumor Necrosis Factor-alpha (TNF-α, Cat. No. ab208348) and Interleukin-1 beta (IL-1β, Cat. No. ab100704) ELISA Kits were purchased from Abcam (Cambridge, UK); Aspartate Aminotransferase (AST, Cat. No. C010-2-1) and Alanine Aminotransferase (ALT, Cat. No. C009-2-1) Kits were purchased from Jiancheng Bioengineering Institute (Nanjing, China); Superoxide dismutase (SOD, Cat. No. G4306) and glutathione peroxidase (GSH-Px, Cat. No. G4310) Kits were purchased from Servicebio (Wuhan, China); NEBNext^®^ Ultra™ RNA Library Prep Kit and NEB Fragmentation Buffer were purchased from Illumina^®^ (California, USA); 26616/SM1811 marker was purchased from Thermo Fisher Scientific (Massachusetts, USA).

### 2.2 Experimental animals and establishment of the stress-induced liver injury model

Kunming male mice, weighing 18–22 g, 30 animals in total, were purchased from Southern Medical University. The animal production license number is SCXK(Yue)2021-0041, and the animal use license number is SYXK(Yue)2022-0125. This study was approved by the Animal Ethics Committee of Guangdong Pharmaceutical University (Approval No. gdpulacSPF2022317). After a 7-day acclimatization period, the animals were subjected to the experiment.

Based on the dosing range of 20–80 mg/kg of CBD in animal studies (Dopkins et al., 2021; Umpierrez et al., 2022), this study selected 20 mg/kg as the administration dose, with oral gavage given three times daily. After a 7-day acclimatization period, the Kunming mice were randomly divided into three groups: Control group, Model group, and CBD group, with 10 mice in each group.

**FIGURE 1:**
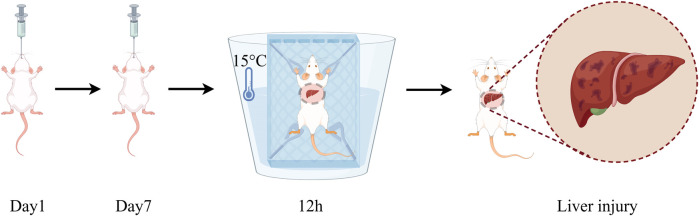
Schematic diagram of monitoring timeline for drug therapy and model establishment.

The establishment of the water immersion restraint stress model was based on the method reported by Zhang et al. (2022), with appropriate modifications. In brief, after 12 h of fasting with free access to water, mice in all groups except the Control group underwent the water immersion restraint stress procedure. The procedure is as follows: the mice were restrained by binding their limbs to a metal mesh and placed in cold water at 15°C ± 2°C, with the water level reaching approximately the neck of the mice. The mice were kept in a room maintained at 20°C with air conditioning, and the stress exposure lasted for 12 h (Figure 1).

### 2.3 Measurement of serum CORT, TNF-α, IL-1β, AST, and ALT levels

After anesthetizing the mice with isoflurane, blood was collected from the mice’s eyes. Blood samples were allowed to stand at room temperature for 30 min and then centrifuged at 1500 g for 15 min at 4°C to separate the supernatant. The measurements were performed according to the instructions provided with the ELISA kits for each parameter. The main steps included: dilution of the standard samples, sample addition, enzyme addition, incubation, washing, color development, termination, and measurement.

### 2.4 Measurement of SOD and GSH-Px in liver tissue

Liver tissue (20 mg) was accurately weighed, and 180 μL of physiological saline was added. A 10% homogenate was prepared under cold conditions, followed by centrifugation at 4000 g for 10 min (Pang et al., 2023). The supernatant was collected and the SOD levels were measured using the Sevier SOD and GSH-Px kits according to the manufacturer’s instructions.

### 2.5 Hematoxylin and Eosin (HE) staining of liver tissue

Liver tissue from the mice was fixed in 4% paraformaldehyde solution (Wuhan Sevier Bio, China) for 12 h. The tissue was then washed, dehydrated, cleared, infiltrated with paraffin, embedded, sectioned (3–5 μm thick), repaired, baked, stained, and sealed to obtain HE-stained liver tissue sections (Ma et al., 2022).

### 2.6 Immunohistochemistry (IHC)

Paraffin sections were treated with 3% H_2_O_2_ solution at room temperature for 5–10 min to eliminate endogenous peroxidase activity. After blocking with 5% BSA for 10 min, the serum was discarded, and CB_2_R antibody (Cat. No. bs-2377R, Bioss China) were applied and incubated overnight at 4°C. After three washes with PBS (5 min each), a secondary antibody was added and incubated at room temperature for 1 h. The sections were then washed with PBS, stained with DAB, and finally sealed and photographed (Jiayao et al., 2023).

### 2.7 Transcriptomic analysis

#### 2.7.1 Total RNA extraction

Approximately 200 mg of liver tissue was washed 2-3 times with RNAse-free PBS and homogenized in TRIzol solution. After standing for 10 min, chloroform was added and the mixture was centrifuged at 13400 g at 4°C for 15 min. The upper aqueous phase was collected and mixed with an equal volume of isopropanol, followed by 10 min of incubation at room temperature and centrifugation. The resulting RNA pellet was collected. RNA purity was assessed using a NanoPhotometer (OD260/280 and OD260/230 ratios), and RNA concentration was quantified with Qubit 2.0. RNA integrity was assessed with an Agilent 2100.

#### 2.7.2 cDNA library construction and transcriptome sequencing

After ensuring the RNA quality met the requirements, the NEBNext^®^ Ultra™ RNA Library Prep Kit (Illumina) was used to construct the cDNA library. The process included RNA separation, fragmentation, reverse transcription, purification, adapter ligation, selection of ∼200 bp cDNA fragments, PCR amplification, and purification. The library was quality-checked, and sequencing was performed on an Illumina Novaseq 6000.

#### 2.7.3 Differential gene analysis

Raw data was filtered, indexes were built, and differential gene expression was analyzed between groups by setting thresholds for differential expression. Differentially expressed genes were selected and subjected to KEGG and GO enrichment analysis to determine enriched pathways and gene functional classifications.

### 2.8 Transmission electron microscopy

Under anesthesia, a small piece of liver tissue was quickly excised using sterilized scissors and placed into a culture medium containing fixative (glutaraldehyde) within 2 min. The tissue was then fixed, dehydrated, polymerized, and ultrathin sections were prepared. The sections were examined and photographed under a transmission electron microscope.

### 2.9 Western blotting

Liver tissue was lysed in lysis buffer (Cat. No. P0013B, Beyotime, China) containing protease inhibitors (Cat. No. P1005, Beyotime, China) using a tissue homogenizer. In brief, proteins were diluted in loading buffer (Cat. No. P0015, Beyotime, China), denatured at 100°C for 5 min, and separated by SDS-PAGE (Cat. No. P0012A, Beyotime, China). The proteins were transferred to polyvinylidene fluoride (PVDF) membranes, which were incubated with 5% BSA (Cat. No. V900933, Sigma-Aldrich, USA) for 90 min. Membranes were then incubated overnight at 4°C with the following primary antibodies, followed by incubation with corresponding HRP-conjugated secondary antibodies. The immunoreactive proteins were detected using an ECL detection system.

The dilutions of the primary antibody used in this experiment are as follows: polyclonal rabbit anti-CB2R (CB2R, Cat. No. bs-2377R) antibody was purchased from Bioss (Beijing, China); polyclonal rabbit anti-α-SMA antibody (Cat. No. WL02510) was purchased from Wanlei (Shenyang, China); polyclonal rabbit anti-SLC7A11 antibody (Cat. No. CY7046) was purchased from Abways (Shanghai, China); polyclonal rabbit anti- Achaete-scute complex like 4 (ACSL4, Cat. No. MA531543) antibody was purchased from Thermo Fisher Scientific (Massachusetts, USA). GAPDH antibody (Cat No. 10494-1-AP), β- ACTIN antibody (Cat No. 66009-1-Ig), HRP-conjugated Goat Anti-Rabbit IgG (Cat No. SA00001-2) and HRP-conjugated Goat Anti-Mouse IgG (Cat No. SA00001-1) were purchased from Proteintech (Wuhan, China).

### 2.10 Statistical analysis

Experimental data were analyzed using GraphPad Prism 9.0 software. One-way ANOVA was followed by Tukey’s *post hoc* test to compare the differences between groups, and results are expressed as mean ± standard error of the mean (Mean ± SEM). A *p*-value ≤ 0.05 was considered statistically significant.

## 3 Experimental results

### 3.1 CBD significantly reduces the levels of CORT, AST, ALT, TNF-α, and IL-1β in stress-reaction mice

**FIGURE 2:**
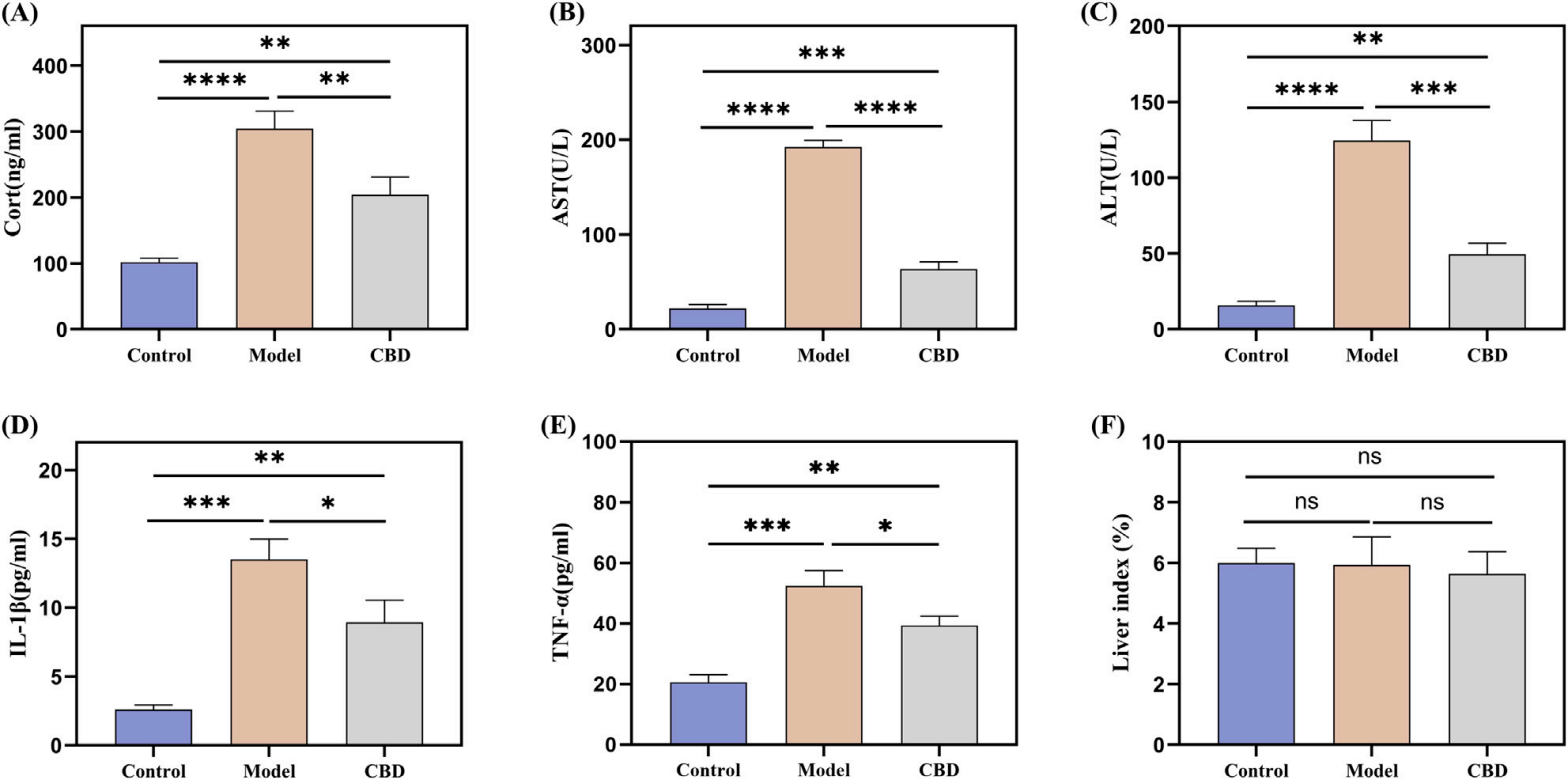
Effect of stress on serum CORT, TNF-α, IL-1β, AST and ALT levels in mice. **(A)** Changes in serum levels of CORT in mice in response to stress; **(B, C)** Changes in serum levels of AST and ALT in mice in response to stress; **(D, E)** Effect of stress on serum contents of pro-inflammatory factors TNF-α and IL-1β in mice; **(F)** Liver index in each group of mice. Mean ± SEM. n = 10. *p < 0.05, **p < 0.01, ***p < 0.001, ****p < 0.001.

The serum biochemical indicators of the mice are shown in Figures 2A–E. The HPA axis is a key system for the body’s response to stress and plays a critical role in physiological stress responses. CORT, a glucocorticoid hormone secreted by the HPA axis under stress conditions, is an important indicator for assessing stress (Fraser et al., 2010; Balasamy et al., 2024). Compared to the Control group, the CORT level in the Model group was significantly elevated (*p* < 0.001), indicating that the stress model was successfully induced. After CBD treatment, CORT production was significantly reduced (*p* < 0.001). AST and ALT are commonly used markers for liver injury. In the Model group, the levels of AST and ALT were significantly increased (*p* < 0.001), suggesting that the stress model induced liver injury in the mice. After CBD treatment, the levels of AST and ALT were significantly reduced compared to the Model group (*p* < 0.001), indicating that CBD has protective effects in reducing liver injury. Furthermore, the levels of TNF-αand IL-1βwere significantly elevated in the Model group (*p* < 0.001). CBD treatment significantly reduced the levels of both TNF-α and IL-1β. Additionally, no significant differences were observed in the liver-to-body weight ratios among the groups (Figure 2F), suggesting that CBD, at the dosage used in this study, has a relatively high safety profile.

### 3.2 CBD alleviates disruption of hepatocyte arrangement induced by stress and effectively improves stress-induced liver injury

**FIGURE 3:**
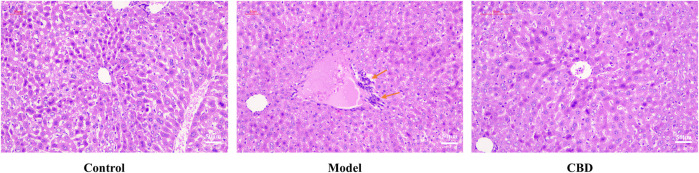
HE staining of mice liver tissue (100X, orange arrows: inflammatory cell infiltration).

HE staining is a primary method for observing morphological changes in tissues. As shown in Figure 3, the liver cells in the Control group exhibited normal morphology and size, with hepatocytes and liver cords arranged radially around the central vein, without significant pathological abnormalities. In the Model group, hepatocyte arrangement was disorganized, with cellular stacking and inflammatory cell infiltration visible in the central region of the liver lobules. After CBD intervention, the infiltration of inflammatory cells was reduced, and hepatocyte morphology and size were restored to near normal, with hepatocytes arranged radially around the central vein. These findings demonstrate that CBD effectively improves stress-induced liver injury.

### 3.3 Transcriptomic results

#### 3.3.1 Differential gene expression between model and control groups

**FIGURE 4:**
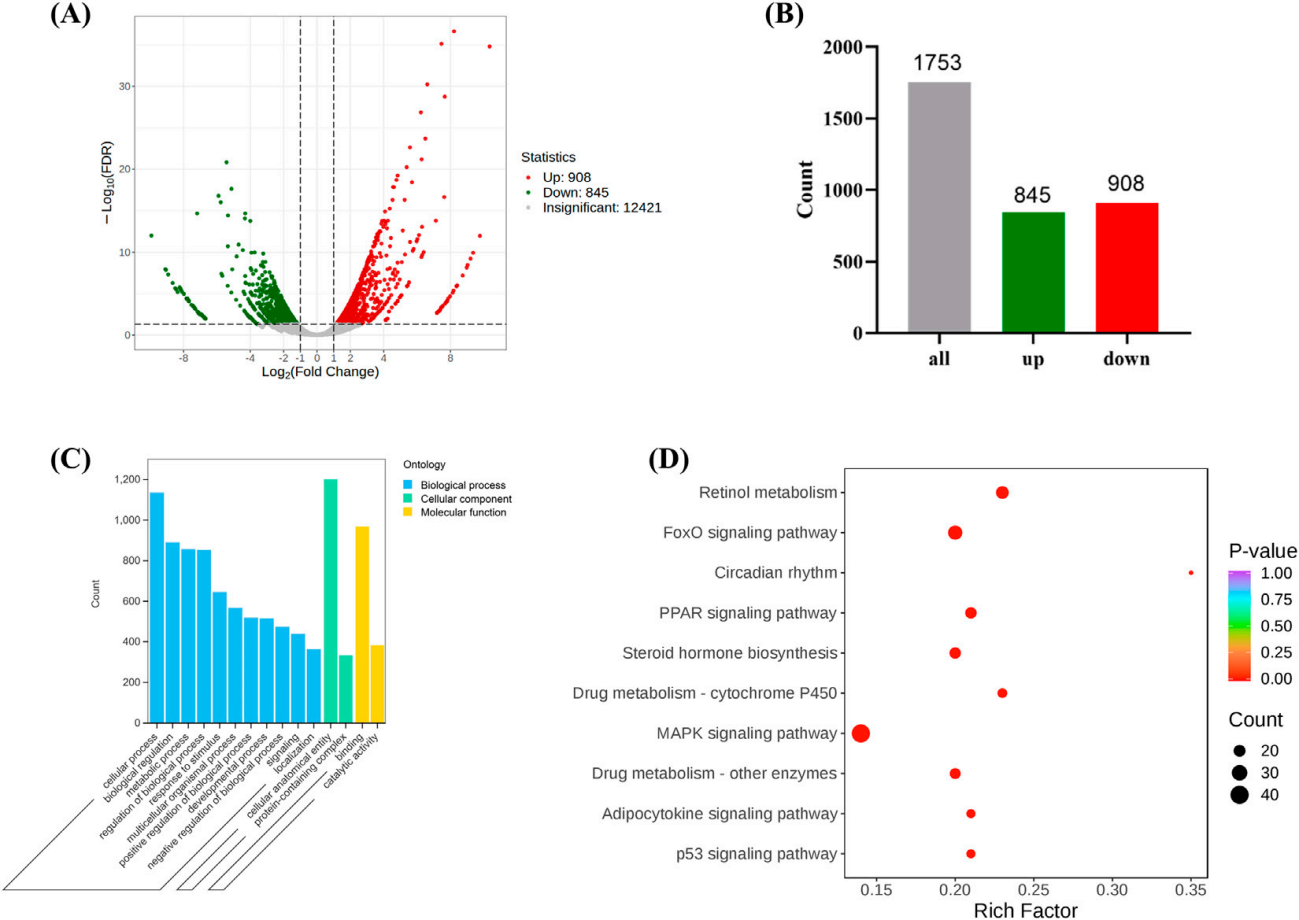
The expression of differentially expressed genes between the Model and the Control. (**(A, B)** are volcano and bar charts of differentially expressed genes, **(C, D)** are GO and KEGG annotation analysis of differentially expressed genes. (Model VS. Control).

Through transcriptomic sequencing, differential expression genes were identified based on the criteria of |log2Fold Change| ≥ 1 and FDR < 0.05. A volcano plot and bar chart were generated to visualize the results (Figures 4A, B), where green and red represent downregulated and upregulated genes, respectively, and gray represents genes without significant expression differences. The results indicated that there were a total of 1,753 differential expression genes between the Model and Control groups, with 908 genes being upregulated and 845 genes being downregulated (Figure 4B). Gene Ontology (GO) analysis revealed that the target genes were mainly enriched in biological processes such as “Cellular process,” “Biological regulation,” and “Response to stimulus” (Figure 4C). These genes also accounted for a significant proportion in the molecular function of “Catalytic activity,” suggesting that they might be involved in antioxidant stress responses and the regulation of mitochondrial functions. Kyoto Encyclopedia of Genes and Genomes (KEGG) pathway analysis identified several significantly enriched pathways, including the MAPK signaling pathway (Wang et al., 2023), p53 signaling pathway (Liu and Gu, 2022) and PPAR signaling pathway (Qu et al., 2021) (Figure 4D), all of which are closely associated with oxidative stress and ferroptosis. In addition, pathways related to circadian rhythm and drug metabolism (Cytochrome P450) may also indirectly affect mitochondrial metabolism and redox balance (Méndez et al., 2016), indicating that oxidative stress plays a crucial role in the development of stress-induced liver injury.

#### 3.3.2 Target gene set selection and pathway enrichment analysis

CBD exhibits a wide range of pharmacological activities that can lead to changes in the expression of multiple genes, consequently affecting the expression of various proteins. This study primarily aims to investigate whether CBD has pharmacological effects and the underlying mechanisms in a stress-induced liver injury model. While other pharmacological effects are not within the scope of this study, it is necessary to narrow down the broad spectrum of differential gene expression caused by CBD administration and select only those genes relevant to this research. The selection of these genes allows for a more precise identification of the specific targets of CBD in treating stress-induced liver injury.

**FIGURE 5:**
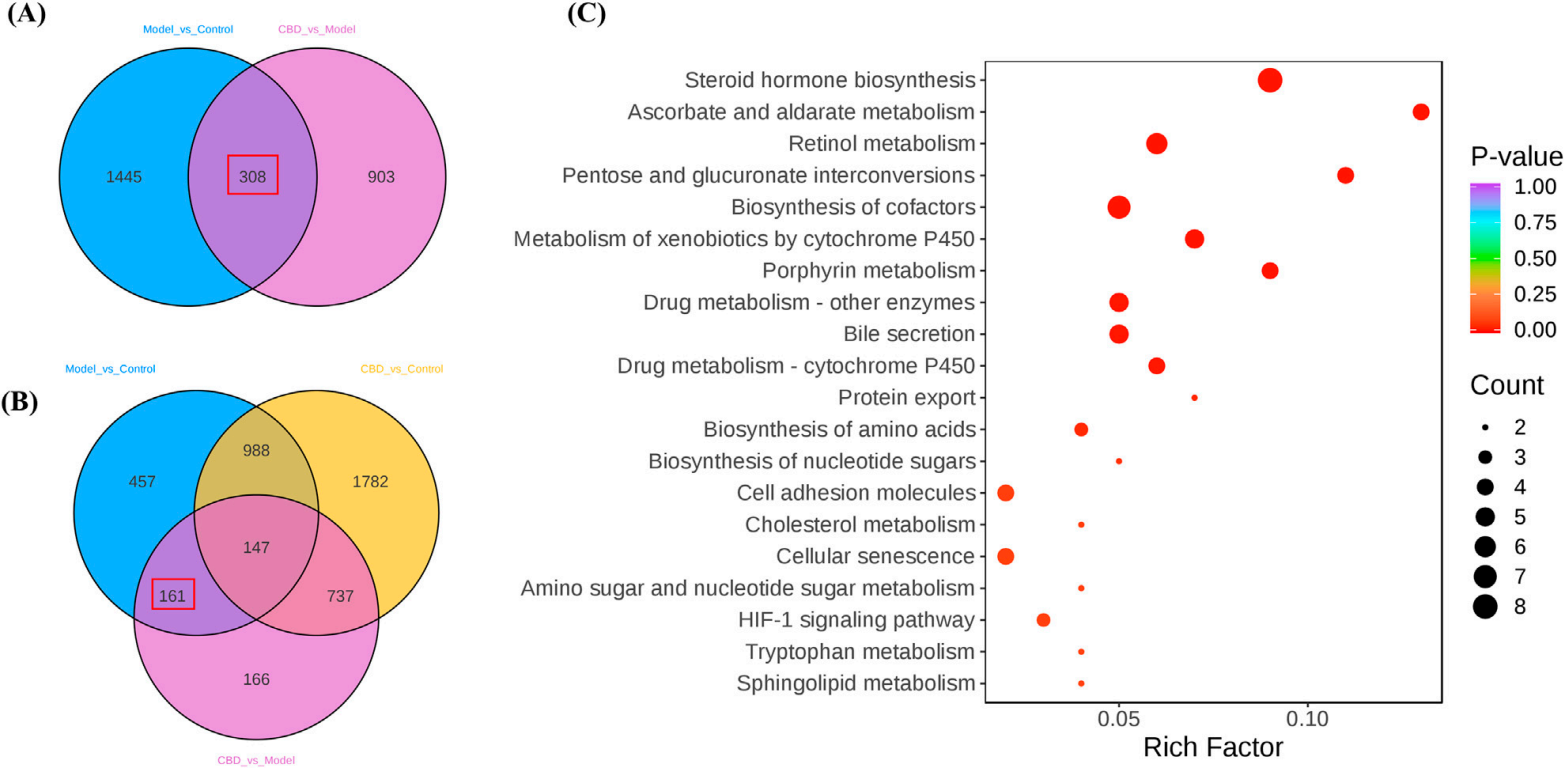
The differential gene screening results of the target genome. **(A, B)** are Venn plots comparing each group, and **(C)** is a bubble plot of KEGG enrichment analysis results for the target gene set).

Using a Venn diagram, 308 differential expression genes related to stress-induced liver injury were selected (target 1, Figure 5A). This gene set includes 161 target genes (target 2, Figure 5B), which share characteristics with target 1 but do not show significant differences compared to the Control group. These target genes represent an initial selection of potential drug targets, which are involved in CBD’s pharmacological action against stress-induced liver injury without causing adverse effects. This gene set serves as the key focus for further investigation in this study.

KEGG pathway enrichment analysis of the target genes (target 2) revealed multiple pathways closely related to mitochondrial metabolism (Figure 5C). Specifically, pathways such as steroid hormone biosynthesis (Papadopoulos and Miller, 2012), retinol metabolism (Walker et al., 2023). Metabolism of xenobiotics by cytochrome P450 and porphyrin metabolism (QF, 2022) all involve key mitochondrial metabolic functions. Additionally, these metabolic processes are often associated with the generation of reactive oxygen species (ROS), which significantly affect oxidative stress levels. The enrichment analysis suggests that CBD may regulate mitochondrial metabolism and oxidative stress to exert its protective effects against stress-induced liver injury.

### 3.4 CBD activates CB_2_R and reduces α-SMA expression to alleviate stress-induced liver injury

**FIGURE 6:**
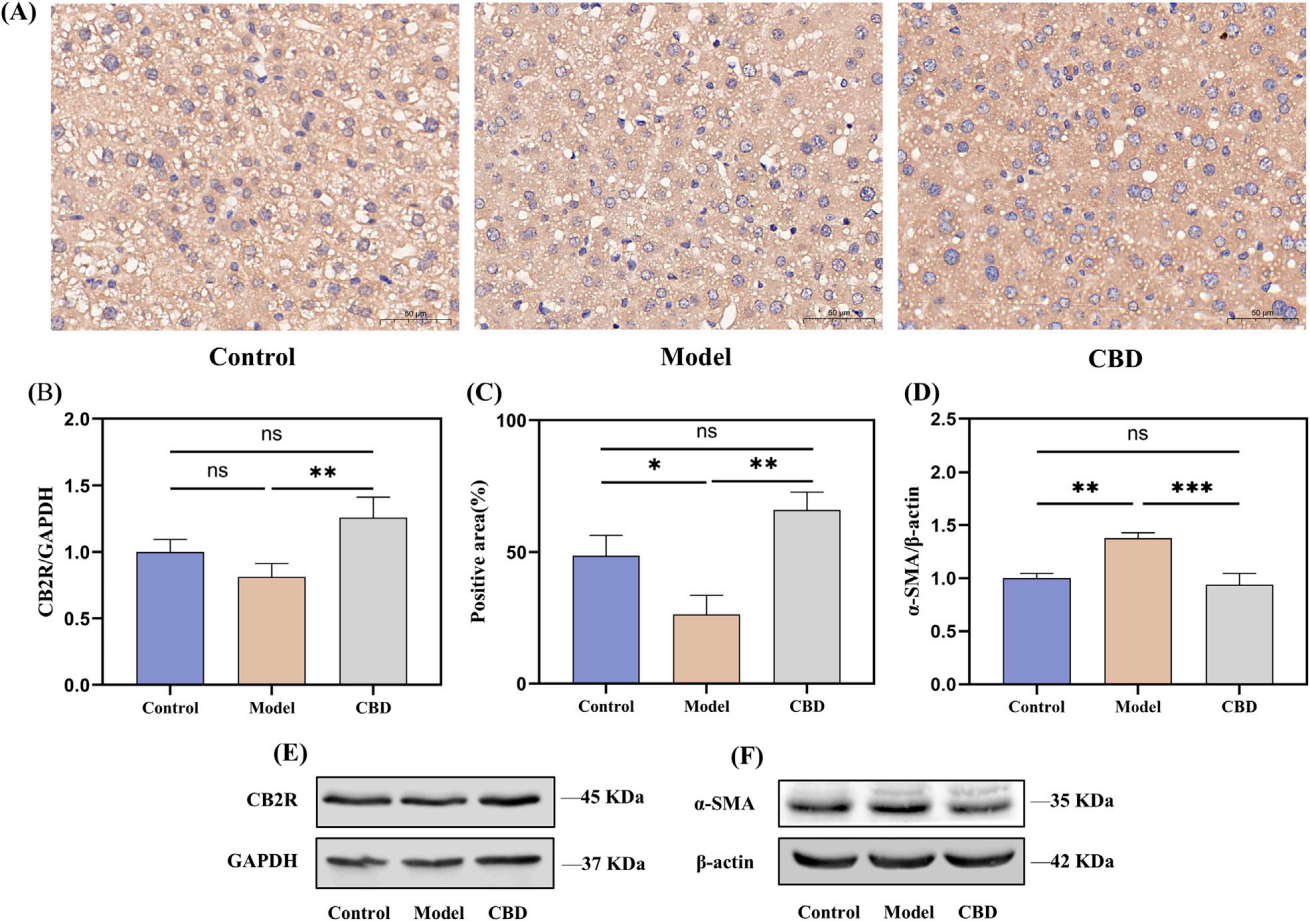
CBD effects on CB_2_R expression in liver tissue following injury. (**(A, C)** immunohistochemical analysis showing the expression of CB_2_R in liver tissue. **(B, D–F)** Western blot analysis of CB_2_R and α-SMA expression in liver tissue).

The immunohistochemical results, as shown in Figures 6A, C, indicated that the expression level of CB_2_R in the model group was significantly reduced (*p* < 0.05) compared to the control group, which was reflected in lighter positive staining, whereas after CBD treatment, the expression of CB_2_R was significantly increased (*p* < 0.05) in the CBD-treated group, as evidenced by the deeper brown staining in the positive regions, which was further corroborated by the Western blot analysis (Figures 6B, E), showing similar trends.

α-SMA is a marker of hepatic stellate cells (HSCs) activation (Le et al., 2023). Following liver injury, HSCs become activated and transform into myofibroblasts, participating in the fibrosis process through the expression of α-SMA. To investigate this, we evaluated the expression level of α-SMA in liver tissues using Western blot analysis (Figures 6D, F). The results showed that compared to the control group, the expression of α-SMA in the model group was significantly increased (*p* < 0.01), indicating the activation of HSCs and the progression of fibrosis following liver injury. In contrast, the expression of α-SMA in the CBD intervention group was significantly reduced (*p* < 0.001) compared to the model group, and after CBD treatment, the α-SMA expression level was not significantly different from that of the control group, suggested that CBD effectively inhibits the activation of HSCs and the progression of liver fibrosis.

### 3.5 CBD alleviates mitochondrial damage and exhibits protective effects against stress-induced liver injury by reducing ferroptosis

**FIGURE 7:**
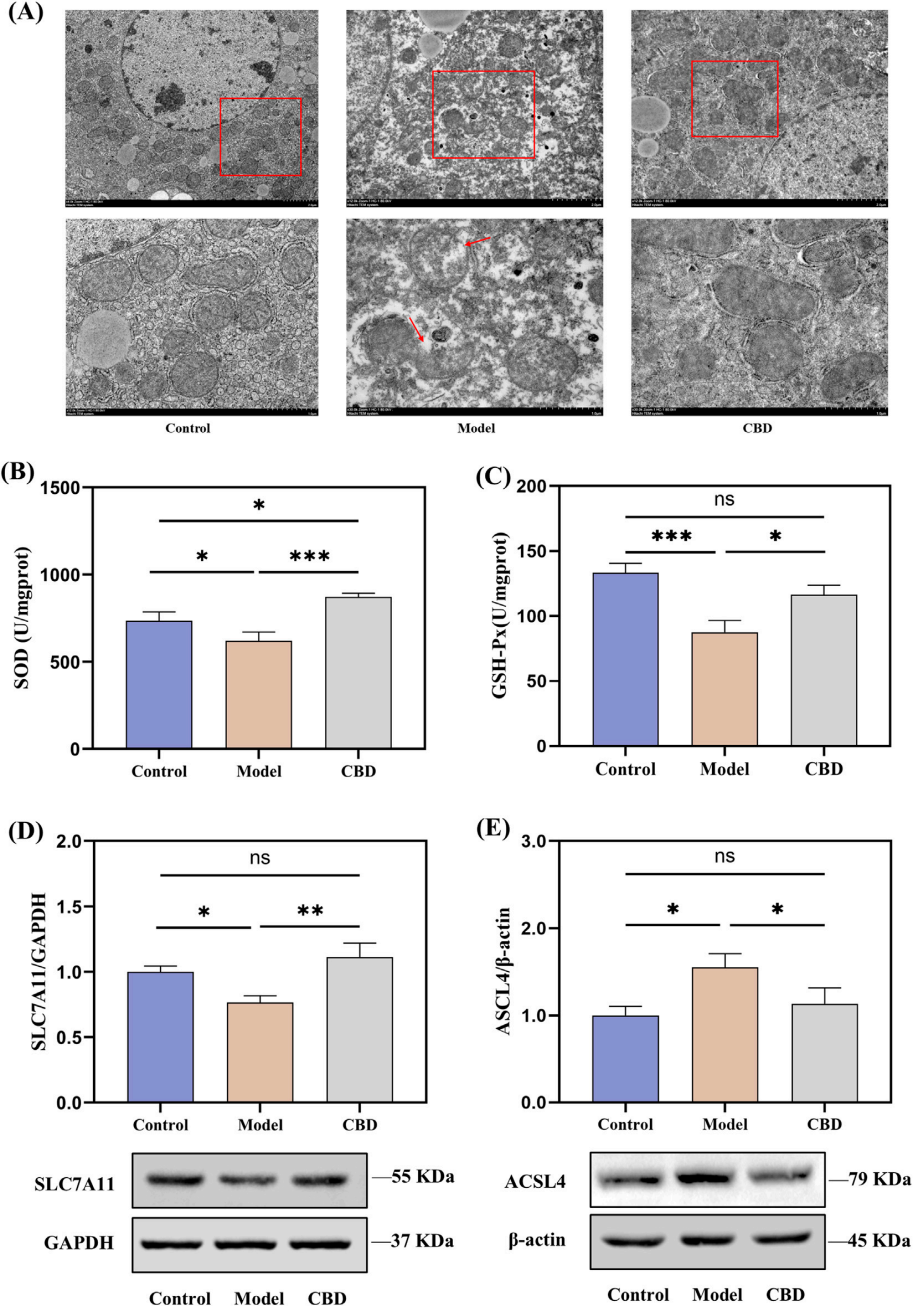
Effects of CBD on mitochondrial morphology, oxidative stress markers, and protein expression in liver tissue (**(A)** TEM images showing mitochondrial morphology. **(B, C)** SOD and GSH-Px levels in liver tissue. **(D, E)** Western blot analysis of SLC7A11 and ACSL4 expression in liver tissue. Mean ± SEM. *p < 0.05, **p < 0.01, ***p < 0.001).

Transcriptomic analysis revealed that the pathways enriched in the target differentially expressed genes were primarily related to mitochondrial metabolism, such as retinol metabolism (Walker et al., 2023), steroid metabolism (Papadopoulos and Miller, 2012) and porphyrin metabolism (Schulz et al., 2023). Therefore, the protective effects of CBD against stress-induced liver injury may be closely associated with mitochondrial function. To verify this, we examined the morphology of liver mitochondria in mice using transmission electron microscopy (TEM) and assessed the levels of SOD and GSH-Px, as well as the protein expression of SLC7A11 and ACSL4. The results are shown in Figure 7.

TEM images revealed that, compared to the Control group, the Model group showed ruptured outer mitochondrial membranes, and the mitochondrial cristae were reduced or absent. CBD treatment significantly improved these mitochondrial morphological changes, bringing them closer to the Control group (Figure 7A). The SOD and GSH-Px results from liver tissues indicated that, compared to the Model group, the levels of SOD (*p* < 0.001) and GSH-Px (*p* < 0.05) were significantly increased in the CBD group (Figures 7B, C). Furthermore, the protein expression of SLC7A11 was reduced in the Model group compared to the Control group (Figure 7D). After CBD treatment, the protein expression of SLC7A11 in the liver tissues of the CBD group was significantly higher compared to the Model group (*p* < 0.05). Additionally, compared to the Control group, the Model group showed a significant increase in ACSL4 protein expression (*p* < 0.05), whereas the CBD group exhibited a marked reduction in ACSL4 protein expression compared to the Model group (*p* < 0.05, Figure 7E). These results suggested that CBD may exert its protective effects against stress-induced liver injury by increasing SOD production, enhancing SLC7A11 protein expression, and reducing ACSL4 protein expression, thereby alleviating mitochondrial damage and mitigating oxidative stress.

## 4 Discussion

It has been reported that 75%–90% of human non-communicable diseases are associated with the prolonged activation of the stress system (Agorastos and Chrousos, 2022). HPA axis is the body’s primary regulatory system for responding to stress (Perry et al., 2019), with CORT serving as a key glucocorticoid secreted by the HPA axis under stressful conditions. CORT levels are an important indicator for assessing the stress state. The cold-water immersion and restraint method is a commonly used stress model to simulate stress responses induced by hunger, cold, and fear of death (Purwoningsih et al., 2022; Zhao et al., 2020). In the present study, the CORT levels in the Model group were significantly increased, confirming that the mice experienced a stress response under the cold-water immersion and restraint conditions. The liver, a vital organ for the detoxification and synthesis of harmful substances in the body, is also one of the most susceptible organs to damage during stress. Therefore, research into the mechanisms and prevention of stress-induced liver injury is of significant importance.

As a cannabinoid receptor, CB_2_R is expressed by various cell types, such as non-parenchymal liver cells, vascular smooth muscle cells, and cardiomyocytes (Tang et al., 2018; Chen J. et al., 2024). Recent studies have shown that activating CB_2_R can exert protective effects by inhibiting inflammation and the fibrotic process (Rakotoarivelo et al., 2024; Matyas et al., 2020), and it also accelerates the regeneration of damaged liver tissue (Teixeira‐Clerc et al., 2010). As an agonist of CB_2_R, CBD can bind to CB_2_R, activate the Gi/oα subunit, and trigger multiple receptor conformations to activate various signaling pathways, thereby affecting cell proliferation, survival, and metabolic regulation (Pongking et al., 2024), playing an important role in liver injury and fibrosis. By activating CB_2_R, CBD inhibits pro-inflammatory signaling pathways such as NF-κB, thereby reducing the production of IL-1β and TNF-α (Chu et al., 2024). The activation of HSCs is a key mechanism in liver fibrosis (Akkız et al., 2024), as HSCs promote fibrosis progression through the excessive expression of α-SMA. Activation of CB_2_R can effectively reduce HSC activation by inhibiting the NOX4 and NF-κB signaling pathway, which in turn lowers the expression of α-SMA and slows down the fibrotic process (Xie et al., 2024). Additionally, studies have shown that CBD can further inhibit HSC proliferation and transformation by modulating the TGF-β/Smad signaling pathway, thus slowing liver fibrosis progression (Fu et al., 2017). This finding is consistent with our results, suggesting the potential of CBD as a liver-protective agent. In conclusion, CBD’s activation of CB_2_R not only exerts anti-inflammatory effects in the liver and extrahepatic tissues but also helps prevent or reverse liver fibrosis, effectively protecting against liver injury.

During the process of screening the target gene set (genes that could induce CBD effects without significant differences from the Control group), we found that the target gene set was primarily enriched in mitochondrial metabolic processes. TEM analysis revealed that the mitochondria in the Model group exhibited disrupted outer membranes, with a reduction or disappearance of mitochondrial cristae. However, this was improved following CBD treatment. The reduction or disappearance of mitochondrial cristae and the rupture of the outer mitochondrial membrane are key features of ferroptosis (Capelletti et al., 2020; Tong et al., 2022). The main causes of ferroptosis are the inactivation of the cellular antioxidant system, disruption of iron homeostasis, and lipid peroxidation (Tang et al., 2021), leading to a decline in antioxidant capacity and the accumulation of ROS, ultimately causing oxidative cell death. To assess the effect of CBD on cellular antioxidant function, we measured the levels of SOD, GSH-Px, SLC7A11, and ACSL4. SOD and GSH-Px are critical antioxidant enzymes that protect cells from oxidative stress damage by catalyzing the conversion of superoxide anions to hydrogen peroxide and clearing hydrogen peroxide and organic peroxides, thereby reducing free radical accumulation and alleviating ferroptosis (Chen T-H. et al., 2024; Contreras-Zentella et al., 2022; Gong et al., 2024). SLC7A11, a gene encoding a cysteine transporter protein, is a key regulator of the “iron overload-ferroptosis” pathway and is involved in cellular antioxidant responses. It prevents the accumulation of lipid peroxides and suppresses ferroptosis (Wang et al., 2017). ACSL4 is positively correlated with ferroptosis, involved in fatty acid metabolism and promoting the production of lipid peroxides, making it an important molecule in the ferroptosis process (Wu et al., 2023). In stress-induced liver injury, the exacerbation of oxidative stress further worsened hepatocyte damage and dysfunction. After CBD intervention, SOD and GSH-Px levels were significantly increased, while the expression of SLC7A11 was upregulated and ACSL4 expression was downregulated. These changes indicated that CBD reduced free radical accumulation, prevented lipid peroxidation, and inhibited iron deposition, thereby alleviating liver damage.

Undoubtedly, CBD exhibited significant protective effects against stress-induced liver injury. However, as this study was solely based on a mouse model and did not assess long-term safety, future research should further explore its efficacy and potential side effects in humans.

## 5 Conclusion

The cold-water immersion restraint method effectively simulates a stress-induced liver injury model caused by conditions such as hunger, cold exposure, and the fear of death. CBD demonstrates protective effects against stress-induced liver injury, and its protective mechanism may be associated with the activation of CB_2_R and mitochondrial metabolism. Specifically, CBD appears to exert its anti-liver fibrosis and antioxidative effects by activating CB_2_R, inhibiting the expression of α-SMA and ACSL4 proteins, and enhancing the expression of SLC7A11 protein, thereby alleviating liver damage.

## Data Availability

The data presented in the study are deposited in the NCBI repository, accession number PRJNA1230758.

## Abbreviations

CBD, Cannabidiol; CB_2_R, Cannabinoid receptor 2; HPA, Hypothalamic-pituitary-adrenal; AST, Aspartate Aminotransferase; ALT, Alanine Aminotransferase; CORT, Corticosterone; TNF-α, Tumor Necrosis Factor-alpha; IL-1β, Interleukin-1 beta; SOD, Superoxide Dismutase; GSH-Px, Glutathione Peroxidase; HSCs, Hepatic stellate cells; ACSL4, Acyl-CoA Synthetase Long-Chain Family Member 4; SLC7A11, Solute Carrier Family 7 Member 11.
